# Combined MSC and GLP-1 Therapy Modulates Collagen Remodeling and Apoptosis following Myocardial Infarction

**DOI:** 10.1155/2016/7357096

**Published:** 2016-11-28

**Authors:** Elizabeth J. Wright, Nigel W. Hodson, Michael J. Sherratt, Moustapha Kassem, Andrew L. Lewis, Christine Wallrapp, Nadim Malik, Cathy M. Holt

**Affiliations:** ^1^Division of Cardiovascular Sciences, Faculty of Biology, Medicine and Health, University of Manchester, Manchester M13 9NT, UK; ^2^Division of Cell Matrix Biology and Regenerative Medicine, Faculty of Biology, Medicine and Health, University of Manchester, Manchester M13 9NT, UK; ^3^KMEB, Department of Endocrinology and Metabolism, University of Southern Denmark, 5000 Odense, Denmark; ^4^Biocompatibles UK Ltd, Chapman House, Farnham GU9 8QL, UK; ^5^CellMed AG, Industriestrasse 19, Alzenau, Germany

## Abstract

*Background. *Mesenchymal stem cells (MSCs) and glucagon-like peptide-1 (GLP-1) are being tested as treatment strategies for myocardial infarction (MI); however, their mechanisms in the heart are not fully understood.* Methods. *We examined the effects of MSCs, either native, or engineered to secrete a GLP-1 fusion protein (MSCs ± GLP-1), on human cardiomyocyte apoptosis* in vitro*. The effect on cardiac remodeling when encapsulated in alginate beads (CellBeads-MSC and CellBeads-MSC + GLP-1) was also evaluated in a pig MI model, whereby pigs were treated with Empty Beads, CellBeads-MSC, or CellBeads-MSC + GLP-1 and sacrificed at one or four weeks following MI.* Results. *MSC + GLP-1 conditioned media demonstrated antiapoptotic effects on ischaemic human cardiomyocytes* in vitro*.* In vivo,* qRT-PCR revealed large changes in the expression of several genes involved in extracellular matrix remodeling, which were altered following MSC ± GLP treatment. After four weeks, infarcted areas were imaged using atomic force microscopy, demonstrating significant alterations between groups in the structure of collagen fibrils and resulting scar.* Conclusions. *These data demonstrate that MSCs ± GLP-1 exhibit modulatory effects on healing post-MI, affecting both apoptosis and collagen scar formation. These data support the premise that both MSCs and GLP-1 could be beneficial in MI treatment.

## 1. Introduction

Myocardial infarction (MI) is one of the leading causes of death in the developed world. Although advancements in the treatment for MI have led to improved survival rates, this has also led to an increase in the incidence of heart failure [1]. The extent of ischaemia-induced apoptosis of cardiomyocytes determines the size of the infarct, which is a key indicator of both survival and the degree of left ventricular remodeling and therefore function after MI [1]. The majority of myocardial cell death occurs in the first 24 hours after the insult [2]; therefore inhibiting cell death at an early stage is crucial to limiting the degree of remodeling and improving prognosis.

After the initial cell death in the infarct, collagen is deposited, which is crucial for maintaining the structure of the heart and preventing cardiac rupture [3]. The properties of the scar are extremely important; a thick, dense scar can result in arrhythmias, whereas a thin, disorganized scar can result in rupture [4]. Scar formation is a highly complex and regulated process; modulation of remodeling could, therefore, be beneficial for healing following MI. Collagen levels are tightly regulated by the opposing actions of matrix metalloproteinases (MMPs) which break down collagen and tissue inhibitors of metalloproteinases (TIMPs) which inhibit MMPs. MMPs are activated within ten minutes of coronary occlusion [5] rapidly degrading extracellular matrix. MMP activity is enhanced in the presence of cytokines such as TNF [6].

Current therapies for treatment of MI aim to restore blood supply and to reduce apoptosis in the infarcted area. One antiapoptotic agent that has demonstrated a clinical benefit by controlling the spread of the ensuing cell death is glucagon-like peptide-1 (GLP-1), a natural gut incretin hormone, which regulates blood glucose homeostasis [7]. GLP-1 and its more stable analogue Exendin-4 (Exenatide) have both demonstrated cardioprotective properties following myocardial infarction (MI) in a number of animal and human studies [8–13]. Administration of the GLP-1 analogue Liraglutide similarly showed cardioprotective benefits following MI, reducing caspase-3 activation via the GLP-1R in a mouse model [14].

Another promising treatment option following MI is stem cell therapy. Although originally intended to directly replace the damaged cells within the compromised myocardium, it is now widely accepted that stem cells exert their beneficial effects largely via paracrine secretion of cardioprotective factors [15–17]. Initial outcomes from stem cell trials in the hearts of both animals and humans were promising; however, more recent clinical trial results have shown mixed results [18–20]. Increasingly, MSCs are being modified to enhance their cardioprotective effects through overexpression of key proteins and factors [21–25].

Previous work from our group has demonstrated the cardioprotective effects of MSCs engineered to secrete a GLP-1 fusion protein (CellBead™) in a porcine model of MI [26, 27]. The fusion protein comprises of two GLP-1 molecules adjoined by an intervening peptide, which has the same action as GLP-1 but with an increased resistance to enzymatic breakdown by the enzyme dipeptidylpeptidase-IV (DPP-IV) and thus a longer half-life* in vivo*. These MSCs are also known to secrete a number of other potent cardioprotective factors including vascular endothelial growth factor (VEGF), monocyte chemoattractant protein-1 (MCP-1), interleukin-6 (IL-6), and interleukin-8 (IL-8) [25], resulting in a novel, combined therapy of GLP-1 and paracrine MSC factors.

We previously demonstrated that MSC + GLP-1 treatment following MI had a functional benefit by significantly reducing infarct size four weeks after MI as well as improving cardiac function determined by improved ejection fraction [26]. The benefit was shown to be a result of multiple different mechanisms and we partially attributed the decrease in infarct size to the known antiapoptotic action of the GLP-1 analogue released locally into the infarct zone from the CellBeads. However, there were no significant changes in apoptosis levels between treatment and control groups one week after MI, suggesting that inhibition of apoptosis may occur at a much earlier time point. MSC + GLP-1 treatment also resulted in a significant increase in collagen in infarcted areas; however the underlying mechanisms occurring remain unknown, as is whether this effect is either beneficial or detrimental to the healing process.

The aims of the present study were to further characterize the efficacy of MSC and MSC + GLP-1 treatment in promoting healing and remodeling following MI and the causative mechanisms of action, using both an* in vitro* ischaemia model and a pig embolization model of MI. In order to achieve these aims, we tested the hypothesis that MSCs, either alone or modified to release GLP-1, would

1. decrease apoptosis of ischaemic human cardiomyocytes* in vitro*;
2. alter ECM turnover by influencing the expression of key structural proteins and proteases, which in turn would affect structural remodeling by influencing the macromolecular architecture of fibrillar collagens.

## 2. Materials and Methods

### 2.1. GLP-1 CellBead Production

Primary human mesenchymal stem cells (hMSCs) were obtained from the bone marrow of a healthy, male, 33-year-old donor following informed consent. This procedure was carried out with full ethics approval [28]. The MSCs were immortalized to form a cell line, following stable transduction by a retroviral vector containing human telomerase reverse transcriptase (hTERT), as previously described [28]. Cells were modified to secrete a fusion protein of GLP-1, known as CM1, which exhibits an extended half-life* in vivo* [29]. The cells were then entrapped in alginate beads using a double encapsulation process with ionic crosslinking that can be used to make CellBeads of a minimum 160 *μ*m and up to a maximum 600 *μ*m in diameter (limited by diffusion of nutrients to the core) depending upon their intended use. In this study, we used CellBeads of 600 *μ*m in diameter, consisting of a core of around 3000 cells/bead surrounded by a 20 *μ*m cell-free alginate barrier for immune-protection, as the intention was to administer the beads to induce a coronary infarct. This size of beads is sufficient to lodge in smaller coronary vessels and produce microinfarcts using previously described methods [30]. The amount of CM1 and paracrine factors (VEGF, MCP-1, IL-1, and IL-6), released from the CellBeads, was previously quantified using ELISA as described earlier [25].

### 2.2. *In Vitro* Human Cardiomyocyte Ischaemia-Reperfusion Model

To generate conditioned medium (MEM, 31095, Gibco), cells with (MSC + GLP-1) and without (MSC) GLP-1 expression were cultured for 48 hours (confluent in T25 flask containing 5 mL media), with media replaced after 24 hours. Media were supplemented with L-tyrosine and 10% FCS. Cells were cultured at 37°C with 5% CO_2_. Ischaemic cardiomyocyte apoptosis and viability were then quantified for cells exposed to conditioned media (MSC ± GLP-1), normal MEM media, the GLP-1 analogue Exendin-4 (Ex-4,1933, Tocris Bioscience), and endogenous human GLP-1 (G-9416, Sigma Aldrich).

Adult human primary cardiomyocytes (hCMs) were purchased from Promocell (C-12811) and cultured in specialized myocyte growth medium with supplement mix (C-22270 and C-39275, Promocell). Cells were acquired from donors with informed, written consent and in compliance with appropriate ethical approval. Ventricular cells were pooled from female Caucasian donors aged 31 and 51 years. These myocytes were confirmed as immunopositive for sarcomeric alpha-actinin, GATA-4, and slow muscle myosin and were negative for CD90, confirming their phenotype; the cells, however, do not contract (Promocell, Germany). Myocytes were used for assays between passages 4–7.

An adapted form of the ischaemic pellet method was used, which has been described previously [31, 32]. Cells from two confluent T150 flasks were enzyme dispersed, collected into a falcon tube and centrifuged at 1,200 r.p.m for five minutes to form a pellet. Supernatant was removed and a layer of mineral oil (M5310, Sigma Aldrich) was added to prevent oxygen exchange. The cells were then incubated at 37°C for one hour, resulting in ischaemia. The cells were resuspended in media and seeded at a density of 135,000 cells/mL in a 24-well plate for a trypan blue viability assay or an eight-chamber culture slide for subsequent TUNEL staining. Immediately after seeding, human cardiomyocytes were exposed to either Basal media, MSC conditioned media (MSC-m), MSC + GLP-1 conditioned media (MSC + GLP-1m), media + Exendin-4 (Ex-4), or media + GLP-1 (GLP-1). All peptides were used at a final concentration of 100 nM.

Apoptosis was measured after 24 hours using TUNEL staining, according to the manufacturer's instructions (G3250, Promega). Cells were mounted with Vectashield containing DAPI (H-1500, Vector) and examined under fluorescent light. With the observer blinded to the experimental conditions, three random fields of view were counted using a ×10 objective and apoptotic cells were expressed as a percentage of total cell number (*n* = 8 per condition). After 48 hours of incubation, cells were detached with trypsin and then incubated with trypan blue (T8154, Sigma Aldrich). The percentage of dying cells (blue stained) and total cell number, representing viability, were counted using a standard haemocytometer (*n* = 4).

### 2.3. Pig Embolization Model of Myocardial Infarction

Myocardial infarction was induced in female Yorkshire White pigs using a bead embolization model, as previously described [30]. The left anterior descending coronary artery branches were embolized using either empty alginate beads (Beads), alginate beads containing immortalized human mesenchymal stem cells (CellBeads-MSC), or beads containing MSCs engineered to secrete glucagon-like peptide-1 (CellBeads-MSC + GLP-1), also as previously described [26]. MI was confirmed using echocardiography and ECG. Pigs were allowed to recover and sacrificed at one week or four weeks after intervention (*n* = 3–5 per group). Tissue was collected and either stored in RNAlater solution (Sigma Aldrich) for qRT-PCR analysis or fixed in paraformaldehyde for subsequent tissue processing embedding in paraffin wax prior to sectioning for histology and AFM.

### 2.4. Quantification of mRNA Expression of Genes Involved in ECM Remodeling in Porcine Myocardium

RNA was isolated from porcine tissue removed one week and four weeks after MI. Tissue was taken from three distinct areas: infarct, border, and remote, where border is the tissue immediately surrounding the infarct and remote tissue is healthily distinct from the infarcted area. RNA was converted into cDNA using a high capacity RNA to cDNA kit (Applied Biosystems). The cDNA was amplified using a SYBR green mastermix (Applied Biosystems) and the reaction quantified using a 7900 HT fast real time PCR system (Applied Biosystems), with the sequence: 50°C for two minutes followed by 95°C for 10 minutes; this was then followed by 40 cycles of 15 seconds at 95°C and one minute at 60°C. For dissociation, the samples were then heated to 95°C for 15 seconds and 60°C for 15 seconds. Data was analysed using the Pfaffl method, which allows for comparison of primers with different amplification efficiencies. Efficiencies were calculated using LinRegPCR software with any samples having an amplification efficiency of zero or more than 2.2 being excluded.

The mRNA of the following genes was quantified: TGF-*β*1, TIMP1, TIMP2, MMP2, MMP9, collagen I (Col1a1), and collagen III (Col3a1) and 18S was used as a housekeeping gene (selected using a geNorm kit from Primer Design). All primers were custom made by Primer Design, UK, with the sequences shown in Table 1.

### 2.5. Analysis of Collagen Fibril Orientation and Macromolecular Structure in Porcine Myocardium Using Atomic Force Microscopy

Paraffin wax-embedded infarcted porcine ventricular tissue was classified as either infarct, border, or remote tissue based on H&E staining, as previously described [26]. Serial sections (7 *μ*m) were cut and mounted on poly-L-lysine coated slides. Sections were dewaxed in xylene, rehydrated through a graded alcohol series, and washed in distilled water for five minutes. Sections were left to air dry before AFM analysis. Areas of interest were identified by staining adjacent sections with picrosirius red for one hour before examination under polarized light [33].

Areas that were identified as collagen rich from the picrosirius red staining were imaged using intermittent contact mode in air using a Bruker Bioscope Catalyst AFM with a Nanoscope V Controller, mounted on a Nikon Eclipse Ti-I optical microscope. Imaging was performed using Olympus high aspect ratio etched silicon probes (OTESPA) with nominal spring constant of 42 N/m (Bruker, AXS, Cambridge, UK). The instrument was periodically calibrated using a grating with 180 nm deep 10 mm^2^ depressions. Height and amplitude data was recorded for 10 × 10 *μ*m scans for each animal in each group (Beads, Beads-MSC, and Beads-MSC + GLP-1) with 3–5 animals per group.

Individual collagen fibrils and fibril bundles were visualized in 3D surface representations generated from the height data using WSxM 4.0 software [34]. Mean fibril periodicity was calculated from the fundamental frequency of the power spectral density distribution in amplitude images determined by WSxM software [35]. The inclusion criteria for a fibril were clear D-periods, a minimum length of 800 nm, and a clear fundamental frequency peak in the power spectral density plot. In this way, 15 fibrils were measured per animal (*n* = 3–5 per group, equating to a mean population size of 45–75 fibrils per group). Fibril diameter was measured by drawing a line across each individual fibril. The organization of the collagen was measured qualitatively by examining the 3D structures formed and the alignment of the fibrils.

### 2.6. Statistical Analysis

ANOVA with Bonferroni post hoc analysis was used to test for differences between treatment groups performed in pigs. ANOVA with Dunnett's *t*-test was used to test for differences between different treatment groups and control in the* in vitro* experiments. All data are expressed as means ± SEM. Statistical significance is denoted as follows: ^*∗*^ *P* < 0.05 and ^*∗∗*^ *P* < 0.01.

## 3. Results

### 3.1. MSC + GLP-1 Conditioned Media Have a Beneficial Effect on Viability of Ischaemic Adult Human Cardiomyocytes* In Vitro*

**Figure 1:**
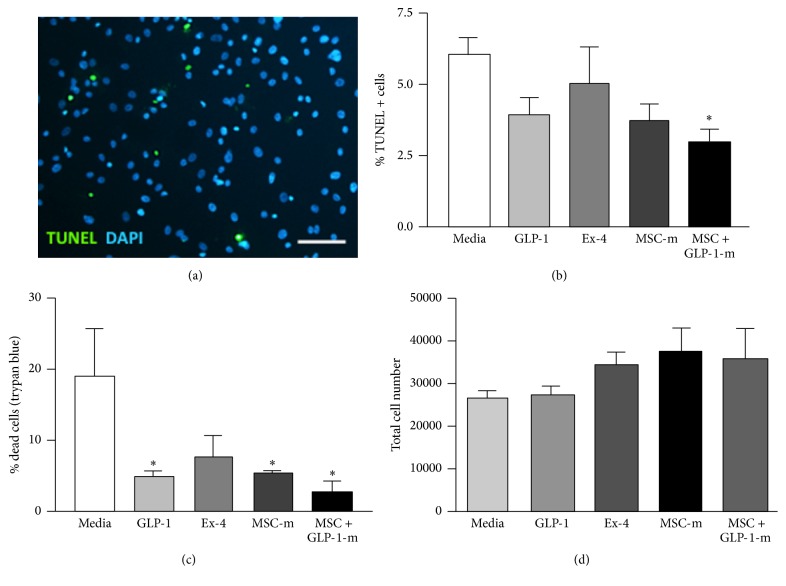
Effect of GLP-1 CellBeads on apoptosis and viability of ischaemic human cardiomyocytes. (a) Apoptosis was assessed using TUNEL staining. Scale bar = 50 *μ*m. (b) 24 hours after ischaemia, MSC-GLP-1 conditioned media significantly reduced apoptosis compared to media alone. GLP-1, Exendin -4, and MSC media showed no significant reduction in apoptosis as assessed by TUNEL. (c) Cardiomyocyte viability was determined using trypan blue dye exclusion assay. GLP-1, MSC conditioned media, and MSC-GLP-1 conditioned media improved cell viability compared to media alone, 48 hours after ischaemia. (d) Ischaemic cell viability determined by cell number was increased with all agonists, compared with media alone; however this was not significant. *N* = 4 per group ^*∗*^ *P* < 0.05 versus media alone.

TUNEL analysis (Figure 1(a)) demonstrated that at 24 hours after ischaemia MSC + GLP-1 conditioned media significantly reduced the number of apoptotic cells compared to media alone (media: 6.14 ± 0.48%; GLP-1: 3.96 ± 0.56%; Ex-4: 5.1 ± 1.22%; MSC media: 3.71 ± 0.56%; MSC + GLP-1 media: 2.97 ± 0.4%, *P* < 0.05, Figure 1(b)). At 48 hours after ischaemia, all agonists apart from Exendin-4 reduced the total number of dead cells, as identified by trypan blue staining, compared to media alone (media: 19.05 ± 6.80%, GLP-1: 5.05 ± 0.54%, Ex-4: 7.88 ± 0.39%, MSC media: 5.41 ± 0.39%, and MSC + GLP-1 media: 2.58 ± 1.65%, *P* < 0.05; Figure 1(c)). Although there was a trend towards increased cell viability (measured as total live cell number) after exposure to all agonists (Figure 1(d)), no significant differences were observed.

### 3.2. Collagen and TIMP Are Upregulated after MI and This Is Modulated following CellBead-MSC and CellBead-MSC + GLP-1 Treatment

**Figure 2:**
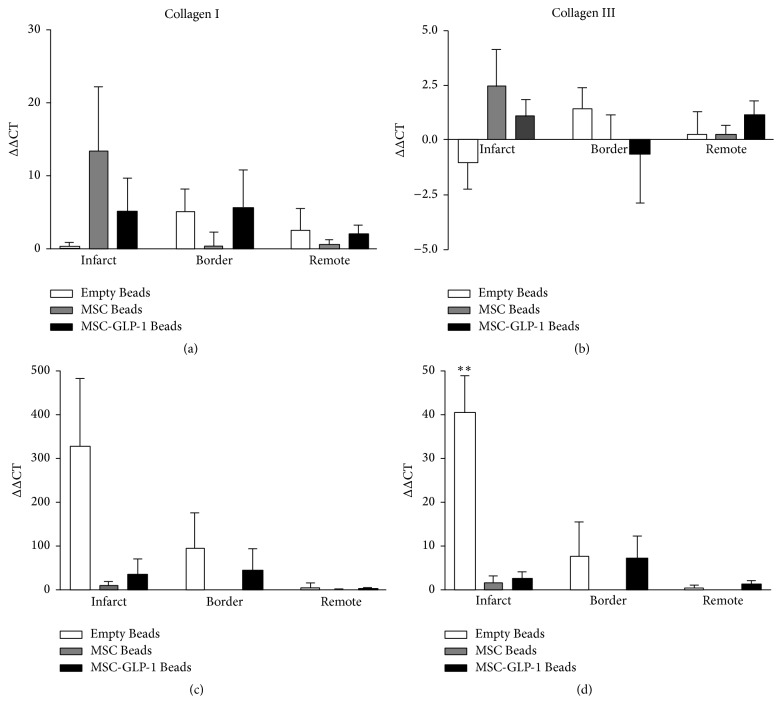
Quantification of collagen I and collagen III mRNA levels. No significant differences were observed between collagen I (a) and collagen III (b) mRNA levels within myocardium from infarct, border, and remote zones at one week after MI. At four weeks after MI, large increases in collagen I (c) and collagen III (d) mRNA levels were observed in the Empty Bead group in both infarct and border regions and this was significantly higher than the CellBead-MSC and CellBead-MSC + GLP-1 treated groups for collagen III. *N* = 3–5 per group; *P* < 0.01.

**Figure 3:**
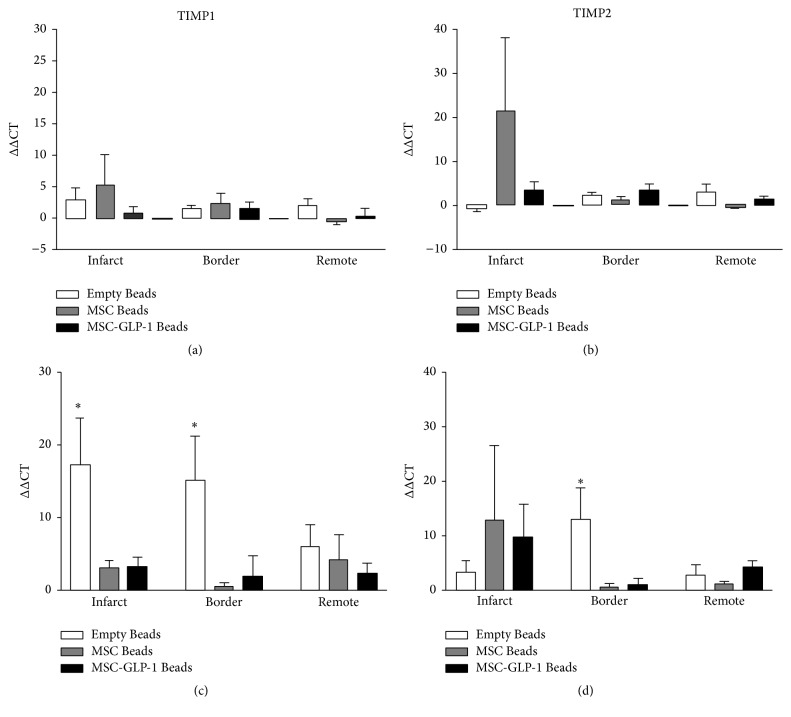
Quantification of TIMP1 and TIMP2 mRNA levels. (a) One week after MI, small increases in TIMP1 mRNA levels were observed. (c) After four weeks, significant increases were seen in both infarct and border regions of the Empty Bead group. (b) After one week, no significant differences were observed in TIMP2 mRNA levels; however four weeks after MI, significantly more mRNA was detected in the infarct and border zone of the Beads group (d). *N* = 3–5 per group; *P* < 0.05.

**Figure 4:**
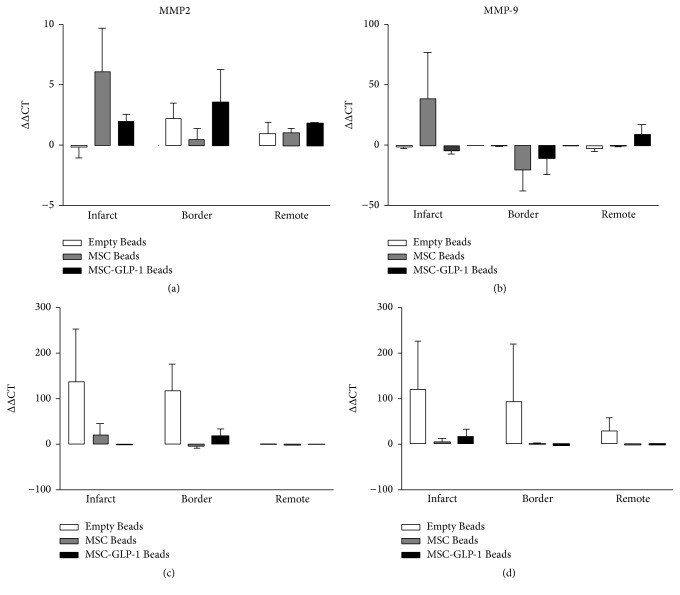
Quantification of MMP2 and MMP9 mRNA levels. No significant differences were observed between MMP2 (a) and MMP9 (b) mRNA levels within myocardium from infarct, border, and remote zones one week after MI. At four weeks after MI, increases in MMP2 (c) and MMP9 (d) mRNA levels were observed in the Empty Bead group in both infarct and border regions of pig heart after myocardial infarction. *N* = 3–5 per group; however these differences were not significant.

At one week after MI, although increases in mRNA expression were observed in all the genes examined in the infarct zone of the Beads-MSC treated group, none of these differences were significant. At four weeks after MI, however, increases in expression of collagen III (Figure 2) TIMP1, and TIMP 2 (Figure 3) were observed in the Beads treated group compared to CellBead-MSC and CellBead-MSC + GLP-1 treated groups; that is, the treatment caused a significant reduction in collagen 3 and TIMP1 mRNA levels four weeks after MI. No differences in collagen and TIMPs mRNA expression were observed following treatment in the remote regions (i.e., distinct from the infarct). Differences in expression are demonstrated in Tables 2 and 3. No significant differences were observed between MMP2 and MMP9 mRNA levels within myocardium from infarct, border, and remote zones one week after MI. At four weeks after MI, increases in MMP2 and MMP9 mRNA levels were observed in the Empty Bead group in both infarct and border regions of pig heart after myocardial infarction. *N* = 3–5 per group; however these differences were not significant (Figure 4).

### 3.3. TGF-*β* Is Increased in the Border Zone after MI and This Is Modulated following CellBead-MSC and CellBead-MSC + GLP Treatment

**Figure 5:**
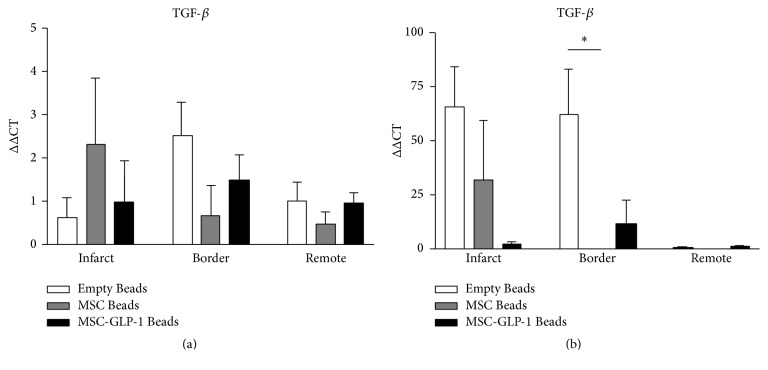
Quantification of TGF-*β* mRNA levels using qRT-PCR. (a) One week after MI, all groups exhibited minor increases in mRNA levels across all regions. (b) Four weeks after infarction, large increases in TGF-*β* were found in the infarct of Beads and Beads-MSC groups. A significant increase was observed in the Empty Bead group in the border zone. *N* = 3–5 per group; *P* < 0.05.

At one week after MI, TGF-*β* MRNA levels were slightly increased over control in all groups; however, by 4 weeks after MI, a significant increase was observed in the border zone myocardium; this increase was not observed in either the CellBead-MSC or CellBead-MSC + GLP-1 treatment groups (Figure 5). No differences in TGF-*β* mRNA expression were observed following treatment in the remote regions (i.e., distinct from the infarct). Differences in expression are demonstrated in Tables 2 and 3.

### 3.4. MSC Treatment Alters Collagen Scar Structure Four Weeks after MI

**Figure 6:**
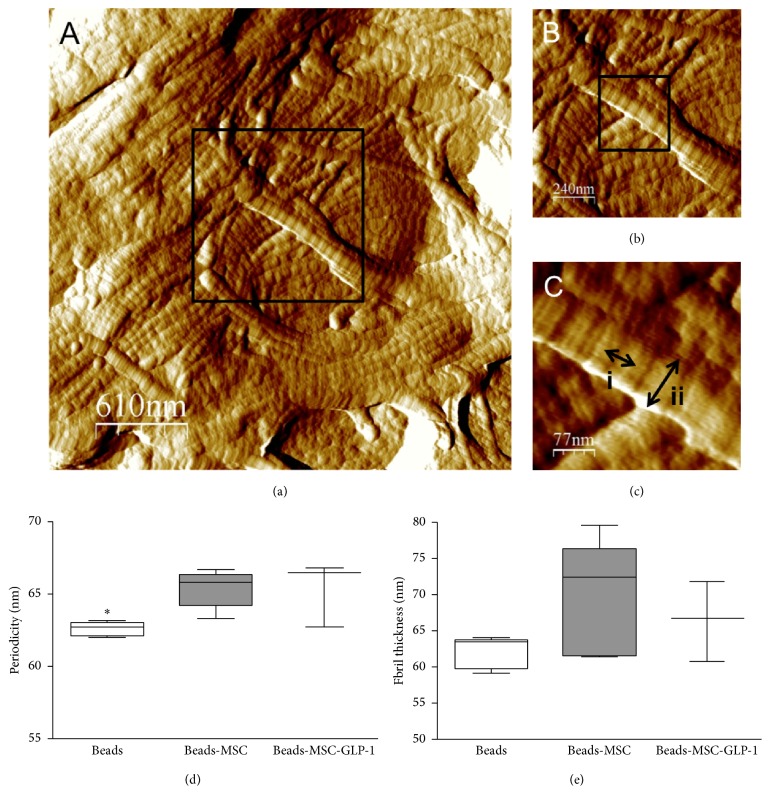
Collagen fibril structures identified using AFM. (a) Fibre bundles from an untreated scar four weeks following MI. (b) A higher magnification of micrograph (a) reveals more details of the individual fibrils comprising the bundle, with D-periods clearly visible. (c) D-period length (i) and fibril width (ii) are indicated by arrows. There were a significantly increased periodicity (d), *P* < 0.05, and a trend towards increased fibril thickness (e) in MSC treated groups four weeks after MI.

AFM imaging of dewaxed ventricular biopsies revealed detailed structural information about the collagen fibrils, with individual D-periods clearly visible (Figures 6(a)–6(c)) and profound changes in fibrillar architecture evident between the Beads and CellBead-MSC treated groups. The Beads treated group is comprised of many thin unorganized fibrils, whereas the group treated with CellBead-MSC displayed thicker fibrils organized into fibre bundles. The CellBead-MSC treated group displayed the most highly organized structure, in terms of alignment and formation of bundles, with the CellBead-MSC + GLP-1 groups showing less organization

Collagen fibril periodicity represents the structure of the fibril. The helical arrangement of the collagen molecules in the fibril creates overlapping bands of polar and nonpolar regions, with the distance between the bands being the D-period. Periodicity was significantly increased in both CellBead-MSC and CellBead-MSC + GLP-1 treated groups (62.15 ± 0.59 nm versus 64.82 ± 0.85 nm versus 65.59 ± 1.64 nm; *P* < 0.05; Figure 6(d)). Fibril thickness/diameter gives an indication of maturity and stability of a collagen scar. Fibril diameter demonstrated a similar trend, with an increase shown in the CellBead-MSC and CellBead-MSC + GLP-1 treated groups compared to the Bead group (62.07 ± 1 nm versus 75.93 ± 4.99 nm versus 66.41 ± 3.17 nm; Figure 6(e)).

## 4. Discussion

Progression to heart failure from myocardial infarction remains a huge problem in the developed world. GLP-1 eluting CellBeads provide a novel therapeutic approach designed to modulate the healing response by localized delivery of cardioprotective factors and have previously been shown to reduce infarct size and improve ventricular functioning in a pig model [26].

In this study, media conditioned with MSCs engineered to secrete GLP-1 significantly reduced apoptosis 24 hours after ischaemia in adult human primary cardiomyocytes. The effect was greater than either GLP-1 or MSC conditioned media alone, indicating a synergistic combined role in promoting cell survival. GLP-1 has been shown to exert antiapoptotic effects on isolated cardiomyocytes [36, 37]; however the effect of GLP-1 or MSC conditioned media on adult human cardiomyocytes has not previously been examined. In addition, by 48 hours after ischaemia, all agonists had significantly reduced total cardiomyocyte death and improved viability compared to media alone. This suggests that GLP-1 exerts its antiapoptotic effects in the early stages following ischaemia and therefore rapid treatment after infarct is required in order to limit infarct expansion. Experiments performed in a pig model of MI have previously demonstrated the beneficial effects of GLP-1 releasing CellBeads on infarct size and myocardial function after MI [26]. The* in vitro* experiments performed in the current study indicate that the factors released from the CellBeads are capable of reducing cardiomyocyte apoptosis. These data provide mechanistic insights into the functional benefit of CellBeads after MI, observed in a pig model of MI.

Myocardial injury following MI triggers an inflammatory response, which initiates the formation of a collagen scar. Levels of mRNA of genes involved in scar formation and the ECM remodeling process were measured in porcine hearts after MI using qRT-PCR. One week after MI, no significant changes were observed in gene expression levels between groups (Empty Beads, CellBead-MSC, and CellBead-MSC + GLP-1). However, the largest increases in gene expression at one week after MI were in the infarct area of the control group treated with CellBead-MSC, which was observed in all genes tested. MSCs are known to secrete a number of molecules that modulate collagen remodeling. Modulated expression of these genes at one week indicates that the remodeling process is initiated at an early stage following MI: TGF-*β* activates myofibroblasts and stimulates the deposition of collagen [38], which was observed in our model as evidenced by increased levels of collagen I and collagen III mRNA. Increased expression of both MMP and TIMP mRNA suggests that remodeling of the ECM is also an ongoing process. GLP-1 appeared to negate the increase in gene expression observed at one week, which could be explained by the previously demonstrated anti-inflammatory effects of GLP-1 [27, 39]. CellBeads-MSC delivered to pig hearts* in vivo* did not reduce infarct size whereas CellBeads-MSC-GLP-1 caused a reduction in infarct size [28] suggesting that the synthesis of collagen and balance of MMPs and TIMPs are finely tuned in the remodeling process.

Four weeks following induction of MI, the significant upregulation of genes in the infarct zone of the Beads treated group indicates a delayed healing response and delayed production of collagen I and collagen III for scar deposition in the Beads treated group. In contrast, in the CellBead-MSC and CellBead-MSC + GLP-1 treated groups, mRNA levels were significantly lower; however gene expression was still increased, indicating that scar formation is not fully completed. Another study investigating the effects of MSCs on ECM-related genes found varying results: I.V. injection of human MSCs into infarcted mice led to decreased fibrosis but upregulation of MMPs and TIMPs [40], suggesting that it is the balance of these two proteins that is an important factor in determining the extent of scar formation. Caution with extrapolation of these qRT-PCR is required as changes in mRNA expression level rather than protein levels are being observed.

AFM analysis allowed for visualization of collagen fibrils and fibres on both molecular and macromolecular scale. Although collagen fibrils have previously been imaged in deparaffinized histological sections of decalcified bone and cartilage [41], to our knowledge, our data demonstrates for the first time that the ultrastructure of the collagen fibril can be clearly imaged in previously paraffin embedded soft tissues. The Beads treated group demonstrated an immature scar phenotype comprised of thin, disorganized fibrils and very few fibre bundles. The CellBead-MSC treated group, however, displayed a mature scar phenotype, with thick fibrils in highly organized fibres. This correlates with the finding of increased expression of collagen, MMPs, TIMPs, and TGF-beta mRNA expression early after MI induction.

Fibrillar collagens are the key mediators of tensile strength in noncalcified mammalian tissues [42] and until recently the periodicity, and hence the molecular structure, of collagen I fibrils was considered to vary between (e.g., 67 nm in rat tail tendon and 64 nm in human dermis), and not within, tissues [43]. Recent AFM based studies performed using our tissue section AFM technique have clearly demonstrated, however, that not only can periodicity vary within a tissue (from 59–66 nm in ovine dermis) but also the suprastructure of collagen fibrils is sensitive to physiologically induced remodeling in oestrogen deprived sheep [44, 45]. Whilst the functional implications of this altered molecular structure remain to be elucidated, it is clear that collagen fibril periodicity may be a sensitive structural biomarker of aberrant tissue remodeling. Further studies are required to decipher the implications of this finding and to see if increased periodicity results in decreased scar stiffness and therefore decreased risk of arrhythmias.

Fibril diameter has previously been used as an indication of structural change [46, 47], with changes in diameter associated with a number of disease states. In this study, collagen fibril diameter was significantly increased in the group treated with MSCs, indicating again that the factors released from MSCs are having a profound effect on the structure of the collagen scar that is deposited following MI. This alteration in the structure would also suggest an alteration in the function of the collagen scar; however further work is required to investigate this.

## 5. Limitations

Due to the highly complex nature of the remodeling process that occurs in the heart after MI, the two time points investigated in this study (one week and four weeks after MI) only provide a limited snap shot of the remodeling process. Future studies should aim to investigate further the mechanisms involved in the remodeling process following MI and the interplay between collagen, MMPs, and TIMPs and apoptosis in regulating this process and how this can be modified therapeutically using MSCs

## 6. Summary

In summary, we have demonstrated that MSCs, either alone or engineered to secrete GLP-1 significantly modulate the healing response following MI. Both paracrine factors from the MSCs and the GLP-1 demonstrated antiapoptotic actions on human adult cardiomyocytes early after ischaemia* in vitro*, with MSCs secreting GLP-1 showing more potent effects. Experiments performed using myocardial tissue obtained following MI in pigs demonstrated that MSCs significantly alter collagen scar formation shown by alterations in gene expression of a number of genes involved in remodeling. This resulted in changes in the myocardium after infarct comprising of fibrils with a higher periodicity and increased diameter. Furthermore we have developed a new AFM technique for high quality imaging of paraffin embedded tissue. Further work is required to examine whether the altered structure of the scar is beneficial for remodeling and recovery following MI.

## Figures and Tables

**Table 1:** Sequences of forward and reverse primers used for qRT-PCR.

Gene	Sense primer	Antisense primer	Product length
MMP2	TTCGACAAGGATGGCAAGTAC	GGAAGCGGAACGGGAACT	100
MMP9	CTTGGTCCTGGTGCTCCT	ATAAGATTGGTTCGTAGGTCTCC	99
TIMP1	AGCCGAAACCTGCACAGT	CACGAGGTTCTTTATTTCATCATCT	80
TIMP2	GCAATGCAGACGTAGTGATC	TCCGTTTGATGGGGTTGC	90
TGFB1	ACTGGGTCTCCGTGTGTTG	ACAAATGAATGGTGGACAGACA	111
COL1A1	ACGGCCTCAGGTACCATGAC	GCTGGGACAGTTCTTGATTTCG	122
COL3A1	TCCCCAAGGTGTCAAAGGT	GTTACCGTTACTGCCAGGAG	103

**Table 2:** Changes in expression levels of genes involved in ECM remodeling one week after MI. Large changes in expression are demonstrated by arrows (↑ = 2–20x increase, ↑↑ = 20–50x increase.

Gene name	Beads			Beads-MSC			Beads-MSC-GLP-1		
	Infarct	Border	Remote	Infarct	Border	Remote	Infarct	Border	Remote
Col1a1		↑	↑	↑			↑	↑	↑
Col3a1				↑					
TIMP1	↑		↑	↑	↑				
TIMP2		↑	↑	↑↑			↑	↑	
MMP2		↑		↑				↑	
MMP9			↓	↑↑	↓↓		↓	↓	↑
TGF-*β*		↑		↑					

**Table 3:** Changes in expression levels of genes involved in ECM remodeling four week after MI. Large changes in expression are demonstrated by arrows (↑ = 2–20x increase, ↑↑ = 20–50x increase, and ↑↑↑ = >50x change). Significance is denoted in brackets.

Gene name	Beads			Beads-MSC			Beads-MSC-GLP-1		
	Infarct	Border	Remote	Infarct	Border	Remote	Infarct	Border	Remote
Col1a1	↑↑↑	↑↑↑	↑	↑			↑↑	↑↑	↑
Col3a1	↑↑ (*∗∗*)	↑					↑	↑	
TIMP1	↑ (*∗*)	↑ (*∗*)	↑	↑		↑	↑		↑
TIMP2	↑	↑ (*∗*)	↑	↑			↑		↑
MMP2	↑↑↑	↑↑↑		↑↑	↑			↑	
MMP9	↑↑↑	↑↑↑	↑↑	↑			↑	↓	
TGF-*β*	↑↑↑	↑ ↑ ↑ (*∗*)		↑↑			↑	↑	
